# Gene Therapy for Mitochondrial Diseases: Current Status and Future Perspective

**DOI:** 10.3390/pharmaceutics14061287

**Published:** 2022-06-17

**Authors:** Alessia Di Donfrancesco, Giulia Massaro, Ivano Di Meo, Valeria Tiranti, Emanuela Bottani, Dario Brunetti

**Affiliations:** 1 Medical Genetics and Neurogenetics Unit, Fondazione IRCCS Istituto Neurologico Carlo Besta, 20126 Milan, Italy; alessia.didonfrancesco@istituto-besta.it (A.D.D.); ivano.dimeo@istituto-besta.it (I.D.M.); valeria.tiranti@istituto-besta.it (V.T.); 2 UCL School of Pharmacy, University College London, 29-39 Brunswick Square, London WC1N 1AX, UK; giulia.massaro.13@ucl.ac.uk; 3 Department of Diagnostics and Public Health, Section of Pharmacology, University of Verona, 37134 Verona, Italy; 4 Department of Medical Biotechnology and Translational Medicine, University of Milan, 20129 Milan, Italy

**Keywords:** mitochondria, mitochondrial DNA, mitochondrial disease, gene therapy, precision medicine

## Abstract

Mitochondrial diseases (MDs) are a group of severe genetic disorders caused by mutations in the nuclear or mitochondrial genome encoding proteins involved in the oxidative phosphorylation (OXPHOS) system. MDs have a wide range of symptoms, ranging from organ-specific to multisystemic dysfunctions, with different clinical outcomes. The lack of natural history information, the limits of currently available preclinical models, and the wide range of phenotypic presentations seen in MD patients have all hampered the development of effective therapies. The growing number of pre-clinical and clinical trials over the last decade has shown that gene therapy is a viable precision medicine option for treating MD. However, several obstacles must be overcome, including vector design, targeted tissue tropism and efficient delivery, transgene expression, and immunotoxicity. This manuscript offers a comprehensive overview of the state of the art of gene therapy in MD, addressing the main challenges, the most feasible solutions, and the future perspectives of the field.

## 1. Introduction

Mitochondria are dynamic double-membrane organelles involved in a myriad of essential cell activities. They are mainly responsible for energy production in the form of adenosine triphosphate (ATP) via the oxidative phosphorylation (OXPHOS) pathway. For this reason, they are considered the cell’s powerhouse [1]. Mitochondria create a dynamic network that is tightly interconnected with other cellular compartments and regulates communication between cells and tissues. They also control various intermediate metabolic pathways, calcium buffering and apoptosis [2], and cellular fitness and genomic integrity [3,4].

Mitochondria have their own genome, which is constituted by several copies of a double-stranded circular DNA molecule (mtDNA), a legacy of their bacterial origins [5]. Only 37 genes are encoded by mammalian mtDNA: 2 ribosomal RNAs, 22 transfer RNAs, and 13 mitochondrial OXPHOS complex proteins [5]. The rest of the mitochondrial proteome, which includes about 1500 proteins, is encoded by the nuclear DNA. For years, it was believed that all mtDNA molecules had the same sequence in physiological conditions, a state known as homoplasmy. When mutations in mtDNA occur, mutant and wild-type molecules cohabit in the same cell/tissue, a condition known as heteroplasmy. However, a deep resequencing analysis recently demonstrated that low-level mtDNA heteroplasmy is extremely frequent in humans, if not a universal finding [6].

Mitochondrial maintenance and homeostasis depend on the coordinated communication between both genomes, and therefore, pathogenic mutations in either the nuclear or mitochondrial DNA result in mitochondrial diseases (MDs), a group of genetic disorders with disrupted OXPHOS. Although considered rare individually, together, MDs are among the most prevalent groups of inherited disorders, with a prevalence of approximately 1 in 4300 (22.9 in 100,000) in adults [7].

A genetic classification of MDs includes two major categories, depending on which genome, mitochondrial or nuclear, carries the responsible mutations. MtDNA mutations have been reported for any gene encoding the OXPHOS subunits, the mitochondrial tRNAs and rRNAs. They include homoplasmic or heteroplasmic point mutations and heteroplasmic large-scale rearrangements. Heteroplasmic mutations cause various clinical manifestations, including mitochondrial encephalomyopathy with lactic acidosis and stroke-like episodes (MELAS) [8], Leigh syndrome (LS) [9], neurogenic weakness, ataxia, and retinitis pigmentosa (NARP) [10], and myoclonic epilepsy with ragged red fibers (MERRF) [11]. The peculiar MD associated with homoplasmic mtDNA mutations is Leber’s hereditary optic neuropathy (LHON) [12]. Mutations in the same gene can result in different clinical presentations; as an example, mutations in the mitochondrially encoded MT-ATP6 gene clinically result in different pathologies, including maternally inherited Leigh syndrome (MILS) [13], NARP [14], and Charcot–Marie–Tooth disease [15], among others. Deletions or duplications of mtDNA have been associated with sporadic progressive external ophthalmoplegia (PEO), Kearns–Sayre syndrome (KSS) [16], and Pearson’s syndrome [17].

Monogenic nuclear mutations have been found in many genes encoding for proteins variously related to the OXPHOS system, including subunits and assembly factors of respiratory complexes, proteins involved in mtDNA maintenance, replication, and translation, and proteins related to mitochondrial dynamics and apoptosis, among others [18].

**Figure 1:**
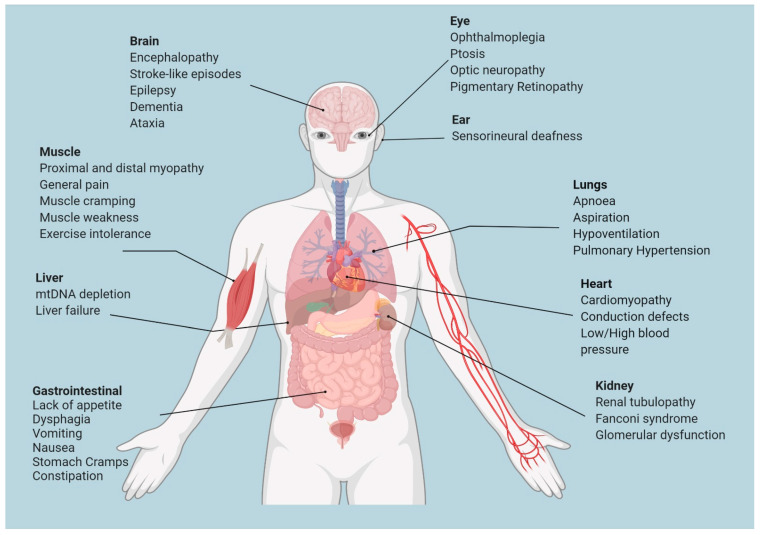
Clinical features of mitochondrial disorder: MDs are caused by oxidative phosphorylation (OXPHOS) failure and display high biochemical, genetic, and clinical complexity, which complicate the prognosis and the development of therapeutic solutions. Adapted from “Adult male” by BioRender.com (accessed on 5 April 2022). Retrieved from https://app.biorender.com/biorender-templates (accessed on 3 June 2022).

MDs are also clinically heterogeneous; they can occur at any age, involving any organ or tissue and manifesting various clinical symptoms [13,19] (Figure 1). The heterogeneity of the clinical manifestation of MDs has strongly limited the diagnosis, management, and development of effective therapies. At present, most of them have no approved cure, and current therapies are focused on relieving complications. However, in recent decades, significant improvements have been reported (please refer to Bottani et al., 2020 for an exhaustive list [20]).

This manuscript will review the gene therapy advances in the treatment of MDs and discuss the challenges and future therapeutic perspectives.

## 2. General Considerations and Technical Aspects

Gene therapy represents an attractive, straightforward therapeutic approach for monogenic recessive diseases. Reintroducing the wild-type form of a mutant gene or other therapeutic genes with the appropriate delivery strategy is now being used in the clinic to treat severe genetic illnesses. While delivering and expressing an ectopic gene throughout the whole body is still challenging, current technologies can target selected cells or tissues to achieve a therapeutic effect.

The concept of gene therapy was first conceived in the 1980s by developing new recombinant DNA techniques. However, genome sequencing and biotechnology advancement have made gene therapy a real medical revolution, offering the possibility to cure many otherwise deadly genetic diseases [21]. The first official gene therapy product of the western world was Glybera, which is to treat lipoprotein lipase deficiency [22]. Many successful clinical trials, mainly using viral vectors, have followed, resulting in the more recent commercialization of Luxturna to treat inherited retinal dystrophy [23] and Zolgensma to treat pediatric patients with spinal muscular atrophy [24].

While most preclinical and clinical studies focus mainly on discrete organs or tissues, such as the nervous system or the eye, the treatment of many MDs would require widespread systemic gene expression that, together with the challenges in delivering genetic material or proteins into the mitochondria, makes developing an effective therapeutic strategy an arduous task.

Different gene delivery approaches, employing either viral or non-viral systems, have been developed for various genetic diseases. Here, we briefly present the relevant technical aspects before focusing on the strategies mainly used to treat MDs.

### 2.1. Non-Viral Approaches

Non-viral gene delivery approaches are based on natural or synthetic compounds (also called non-viral vectors) in which complexes of DNA, proteins, polymers, or lipids are formed in particles capable of efficiently transferring genes into cells by using different methods. Mainly applied for DNA vaccination, physical methods such as the hydrodynamic or ballistic injection of DNA typically employ a physical force to overcome the cells’ membrane barrier and facilitate intracellular gene transfer [25,26] Although physical methods are obviously the simplest and the safest, they showed low gene delivery efficiency and considerable cell damage occurring at the center of the discharge site. Chemical methods include micelles of cationic surfactants, rhodamine nanoparticles, and liposomes, representing the most effective non-viral vectors developed so far [27,28] Cationic liposomes, consisting of microscopic particles with mono- or multi-cationic head groups, are the most used method for gene delivery. They can interact with negatively charged DNA molecules with 100% loading efficiency; nevertheless, an excess of positive charge can harm cell viability. Introducing exogenous DNA into the mitochondria of mammalian cells has the additional difficulty of traversing three membranes to reach the mitochondrial matrix where the mtDNA resides. Fierl et al. demonstrated that oligonucleotides could be successfully introduced into the mitochondria of living mammalian cells by annealing them to peptide nucleic acids coupled to mitochondrial targeting peptides harnessing endogenous import machinery through mitochondrial targeting sequence (MTS)-mediated translocation via the TOMM22/TIMM23 complexes [29] These methods have several advantages over viral approaches, such as the reduced pathogenicity, low cost, and ease of production. Indeed, the primary advantage of using non-viral vectors is their biosafety, but in the context of MDs, many of the non-viral methods are still in the preliminary in vitro stages. They are limited by poor transfection efficiency, weak specificity to mitochondria, or failure due to cytotoxicity [30] Although non-viral approaches have all these potential advantages, at the moment, none of the currently available non-viral vectors fulfil ideal vector properties, and viral gene delivery is the most efficient tool for gene therapy.

### 2.2. Viral Approaches

Viral vector gene therapies use engineered viral delivery systems to introduce specific nucleotide sequences, encoding genes and regulatory RNAs (for example, small interfering RNAs (siRNAs)), into cells.

Classically, a viral vector is defined by the three following components: (1) the protein capsid and/or envelope that encapsidates the genetic cargo and states the vector’s tissue or cell tropism and antigen recognition; (2) the genetic payload of interest, which when expressed in cells, serves to confer the desired effect; and (3) the “regulatory expression cassette”, including enhancer/promoter/auxiliary elements that control the stable or transient expression of the transgene as an episome or as a chromosomal integrant.

Each viral vector differs in designing and manufacturing these three components, has unique considerations, and harbors its strengths and weaknesses. The most commonly used viral vector systems are based on lentiviruses (retroviruses), adenoviruses, and adeno-associated viruses. Each viral vector system possesses certain characteristics that determine its suitability for a specific application [31].

#### 2.2.1. Lentiviral Vectors

Lentiviral vectors (LVs) are recombinant retroviral vectors derived from the human immunodeficiency virus (HIV) with a single-stranded RNA genome converted to double-stranded DNA in the transduced cell by the viral enzyme reverse transcriptase and transported to the cell nucleus. Here, the viral DNA is integrated into the host genome via the viral protein integrase and is consequently replicated and transcribed during the cell cycle. The resultant RNA is transported to the cytoplasm and assembles with structural and replication proteins. New virions are coated with a glycoprotein envelope as they bud from the cellular membrane and are released from the cell [32]. LVs have a large packaging capacity (8–10 kb) and efficiently transduce proliferating and post-mitotic cells, including neural precursors, hematopoietic stem cells, neurons, and glia. Since they integrate into the host cell genome, LV-mediated gene transfer leads to long-term transgene expression in targeted cells [31]. Lentiviral vectors have been engineered to improve their safety profile and limit the potential risk for clinical translation. *First-generation* lentiviral vectors contain a significant portion of the HIV genome, including the viral core, regulatory protein-coding sequences, and accessory regulatory genes [33]. *Second-generation* LVs lack accessory virulence factors such as *vif*, *vpr*, *vpu*, and *nef*, which promote viral proliferation and infection [34]. The removal of the accessory genes does not inhibit the transfer of genetic material to the host cell. In the second-generation Tat-dependent system, the structural protein *Gag* gene; the genes required for reverse transcription, integration, and nuclear export, *pol* and *rev*; and the genes encoding the envelope protein are provided in *trans*. The expression cassette carrying the transgene sequence is flanked by long terminal repeats (LTRs), which mediate integration into the host genome. A more recent *third-generation* of lentiviral vectors further improves safety by splitting the viral gene *Rev* from *Gag* and *Pol* into two different DNA plasmids, making the viral particles replication-incompetent. In addition, part of the 5′-LTR, which contains the TATA box and transcription factor binding sites, can be partially deleted, creating a self-inactivating vector packaging system following integration into the host genome [35]. LVs are mainly used in ex vivo gene therapy, with successful application in the treatment of immune deficiencies [36] and leukodystrophies [37].

#### 2.2.2. Adenoviral Vectors

Adenoviruses (Ad) are non-enveloped viruses that are known to cause infections of the upper respiratory tract and other organs such as the brain and bladder. Ad vectors have an icosahedral protein capsid composed of the structural proteins, hexon, penton base, fiber, capsid protein precursors, and the virion core proteins [38]. The capsid lodges a 26–45 kb double-stranded linear DNA genome. Hairpin-like inverted terminal repeats (ITRs) with variable lengths (30–371 bp at its termini) flank the Ad genome and serve as self-priming structures promoting primase-independent DNA replication [39].

The infection cycle initiates with the interaction between a cell surface receptor, such as coxsackievirus-Ad receptor (CAR), CD46, desmoglein 2 (DSG2), or sialic acid, and the distal domain of the virus capsid fiber [40,41,42,43]. Once the virus is bound to the cell surface, it is then internalized by endocytosis, and the capsid is disassembled in the cytoplasm [44]. The viral DNA then enters the nucleus, passing through the nuclear envelope pore complex [45]. Moreover, exons derived from partially disrupted virions bind to dynein motors to promote the virus traffic to the nuclear pore via the microtubular network [46]. The viral DNA predominantly is not incorporated into the host cell genome; it remains epichromosomal [47].

More than a hundred human Ad genotypes have been recognized and classified into seven species—from A to G—based on a genomic sequence analysis (http://hadvwg.gmu.edu (accessed on 15 April 2022)).

Ad infection in humans typically results in asymptomatic responses or leads to mild or severe disease in immunocompetent individuals. As a result of past infections [48,49], most people carry neutralizing antibodies against one or more of the prevalent human Ad serotypes, which results in lifelong immunity.

Despite Ad vectors having some advantages, such as high transduction efficiency and broad tropism for different tissues, they tend to have compromised potencies [50] and trigger a more robust immunological response than other viral vectors due in part to the epidemiology of Ads in the human population. These aspects limit their use for in vivo gene therapy for monogenic diseases [51] but make them an excellent choice for oncolytic virotherapy [52] and ideal candidates for developing novel vaccines. For example, Ad-based vaccines have been developed against Ebola [53], influenza [54], and the severe acute respiratory syndrome coronavirus 2 SARS-CoV-2 [55] and have been shown to be well-tolerated and induce rapid humoral immune responses. Ads have also been used to deliver vaccines for cancer prevention to induce the expression of oncolytic processes and/or tumor-associated antigens (TAA) that promote antitumor immune responses via Ad-mediated gene delivery.

#### 2.2.3. Adeno-Associated Viral Vectors

Adeno-associated viruses (AAVs), belonging to the *Parvoviridae* family, were first identified in the 1960s as contaminants of adenovirus preparation [56]. AAVs are not linked to any disease in humans or animals and stay episomic in cells for a long time, lowering the risk of insertional mutagenesis and making them popular for gene delivery strategies.

Classified as a dependoparvovirus, AAV requires co-infecting helper viruses (adenovirus or herpes simplex virus), as it needs the essential genes for expression and replication of its genome.

The AAV genome consists of single-stranded (ss) DNA that houses four open reading frames (ORFs) containing the *rep* and *cap* genes involved in DNA replication and capsid formation [57], respectively. The 4.7 kb genome is flanked by 145 nt ITRs that serve as self-priming structures for replication and provide the signal for Rep-mediated packaging. AAV particles bind to or have differential affinities to an array of primary cell surface glycoprotein receptors and secondary receptors [58]. Upon attaching to the cell surface, clathrin-mediated endocytosis is elicited. The AAV unit is then trafficked via endosomal vesicles and transported through the late endosomal–lysosomal compartments. Due to the low pH environment of the lysosomal vesicles, the capsid undergoes conformational changes, escapes the lysosomal compartment, and is then shuttled into the nucleus via nuclear localization signals. The single-strand AAV genome undergoes second-strand synthesis to form the double-stranded (ds) genome configuration required for gene transcription.

AAVs are gaining popularity as a gene delivery tool since they are not linked to any known disease in humans or animals, remain in the episomal state in transduced cells, mediate long-term gene expression, and can transduce different cell types. AAVs are ideal for gene therapy due to their low immunogenicity [59], replication defectiveness, and long-term gene expression [60].

**Figure 2:**
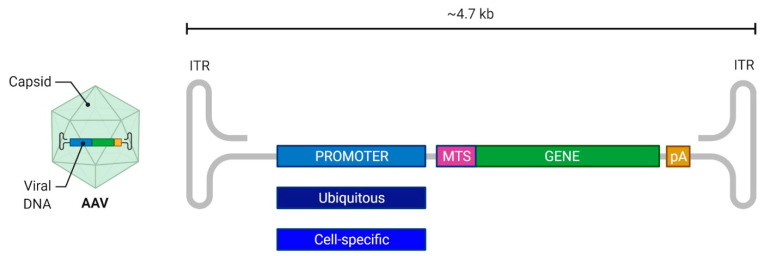
Schematic representation of “Adeno-Associated Virus (AAV) Genome”. Retrieved from https://app.biorender.com/biorender-templates (accessed on 16 April 2022).

Recombinant AAV vectors (rAAV) are generated by replacing the viral ORFs with the transgene cassette flanked by ITRs. The genes involved in replication and capsid assembly are provided in *trans*. Since the packaging size is limited to 4.7 kb (Figure 2), the expression cassette can accommodate only relatively short sequences.

Alternative strategies that exploit ITR-mediated recombination have produced dual-vector systems that can express “oversized” transgenes through transcript splicing across intermolecularly recombined ITRs from two complementary vector genomes [61,62].

A consideration for setting up a gene therapy strategy is that the conversion of single-stranded DNA into double-stranded DNA upon the transduction of target cells represents a crucial time-limiting step in transgene expression from an rAAV vector. Self-complementary AAV (scAAV) vectors have been developed to overcome this limiting step. They are designed as a single-stranded inverted repeats that fold back upon themselves to form double-stranded genomes when entering transduced cells. While *sc* vectors have the advantage of quicker transgene expression, the packaging capacity is halved to >2.5 kb and therefore might not be suitable for big sequences. The specific characteristics of different AAV serotypes can be combined through a pseudotyping process, incorporating the genomic material of one serotype into a different serotype’s capsid [63,64]. Usually, AAV2 ITRs are packaged into other AAV capsids, originating various AAV2/n pseudotyped vectors with improved transduction efficiency and tissue tropism. In addition, the use of pseudotyped vectors based on different serotypes may help evade pre-existing immunity to one specific serotype. Many recombinant vectors have been extensively characterized and are currently used in the clinic. Over 200 rAAV clinical trials have demonstrated excellent safety profiles due to non-integration and episomal persistence for at least ten years [65,66], with two AAV-based drugs currently approved and commercialized.

**Figure 3:**
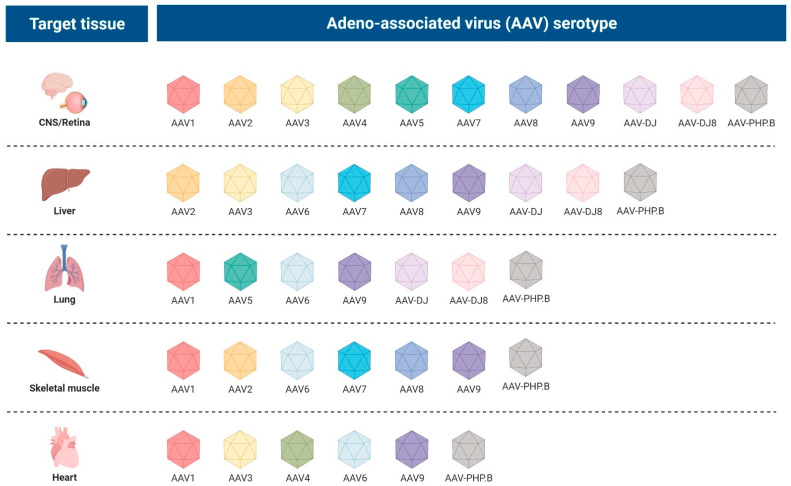
Representation of the different AAV serotypes and their target tissues. Adapted from AAV Tissue Specificity by BioRender.com (accessed on 16 April 2022) (2022). Retrieved from https://app.biorender.com/biorender-templates (accessed on 16 April 2022).

Although promising preclinical and clinical settings have provided critical insights into gene therapy, particularly for neurological disorders, the data suggest that enhanced gene delivery and expression are needed to improve the therapeutic efficacy for long-term treatment. These findings highlight the critical issue of gene therapy vector re-dosing, as gene transfer is currently limited to a single administration. The host immune response to AAV capsid proteins leads to the formation of neutralizing antibodies that prevent other vector administrations. Eleven serotypes of AAV have been identified (Figure 3), and more than 100 AAV variants have been isolated from human/non-human primate tissues and evaluated in various model systems [67]. AAV serotypes 1 to 6, except for AAV5, were isolated as contaminants in laboratory adenovirus stocks, among which AAV2, AAV3, and AAV5 are the ones thought to be of human origin based on the prevalence of neutralizing antibodies in the human population. AAV4 appears to have originated in monkeys since antibodies against AAV4 are common in nonhuman primates [68]. Similarly, AAV10 and AAV11 were isolated from cynomolgus monkeys [69].

Only AAV1–5 and AAV7–9 can be defined as true serotypes. Variant AAV6 does not appear to fit into this definition since its serology is almost identical to AAV1, and the serological profiles of AAV10 and AAV11 are not well-characterized.

Given the specificity of cell types they can infect, i.e., the tropism, AAV serotypes are a beneficial system for preferentially transducing particular cell types. A summary of the tropism of each AAV serotype is reported in Figure 3, which also indicates the optimal serotype (s) for transduction of a given organ.

The AAV2 serotype is the best-characterized and transduces many tissue types, including liver, muscle, lung, and central nervous system (CNS), with moderate efficiency. AAV1 and 5 exhibit higher transduction frequencies within the CNS [64] than AAV2, which shows widespread transduction throughout the entire midbrain, while AAV4, for example, appears to transduce specific cell types resident in the lateral ventricular wall of the brain, the ependyma and astrocytes in the subventricular zone, known for their essential role in adult neurogenesis [70]. It is important to note that the expanded tissue tropism of AAV serotypes is helpful for specific gene delivery applications, but it can result in the transduction of nontarget tissues.

Researchers have further refined the tropism of AAVs through pseudotyping, or the mixing of a capsid and a genome from different viral serotypes, aiming to improve the transduction efficiency and alter tropism. For example, the in vitro transduction of human and mouse primary fibroblasts demonstrated that AAV2/5 and AAV2/8 had higher transduction efficiency rates than AAV2/2 [71].

Scientists have also demonstrated that the viral tropism may be altered using hybrid capsids derived from different serotypes. AV-DJ is an example of a synthetic serotype with a chimeric capsid of AAV-2, 8, and 9. It contains a heparin-binding domain in its capsid, which may efficiently transduce many cell types and escape immune neutralization [72]. It displays a higher transduction efficiency in vitro than any wild-type serotype. The mutant AAV-DJ8 displays the properties of AAV-DJ but with enhanced brain uptake [72].

In particular, specific serotypes of AAV, such as recombinant adeno-associated virus serotype 9 (AAV9) and pseudotype rhesus-10 (AAVrh.10), have shown unique properties to target the CNS in comparison to most AAV serotypes [73], making them a powerful system for delivering genetic material to the CNS since they both share the ability to cross the blood–brain barrier (BBB) [74]. A novel AAV9-derived variant identified by screening a library of AAV9 vectors carrying random mutations at the capsid surface, named AAV-PHP.B, has been shown to cross the barrier far more efficiently, transducing approximately 50–100% of neurons and 80% of astrocytes across multiple CNS regions after peripheral delivery in mice [75]. Deverman et al. also reported that an enhanced AAV-PHP. B variant, named AAV-PHP.eB, shows further improvements in neurons and glia transduction efficiency throughout the CNS after intravenous (IV) delivery in adult mice [76]. Despite the two variants being compared in the rhesus macaque (*Macaca mulatta*) after injection of the same dose [77] and appearing to be a promising research tool, further investigations need to be conducted with additional animals to reach statistical power and to determine the potential implication for their use in clinical trials.

Today, recombinant AAVs (rAAVs) are the leading platform for the in vivo delivery of gene therapies. There are two classes of recombinant AAVs (rAAVs) currently in use: single-stranded AAV (ssAAV) and self-complementary AAV (scAAV). ssAAVs are packaged as either anti-sense (minus-stranded) or sense (plus-stranded) genomes. Such single-stranded forms are transcriptionally inert and must be converted to double-stranded DNA when they reach the nucleus as a prerequisite for their transcription. This conversion can be completed by the host cell DNA polymerases, which synthetize the second strand synthesis, or by the strand annealing of the plus and minus strands that may coexist in the nucleus. Because scAAVs are already double-stranded by design, they can immediately undergo transcription, bypassing the step where a new strand is synthesized [78] and increasing transduction efficiencies compared to ssAAV [79]. However, new engineered serotypes of AAV9, such as PHP.B and PHP.eB, have been developed using a core-based targeted evolution strategy. These new serotypes displayed a 40-fold increased effectiveness in crossing the BBB in C57BL/6 mice compared with its AAV9 ancestor, even when delivered IV [75]. Despite these exciting and promising features, the characteristics of these novel synthetic capsids might not always be translatable to larger animals and non-human primates [76,80]. Furthermore, these variants are of limited clinical utility due to their liver toxicity in non-human animals.

## 3. Gene Therapy for Mitochondrial Diseases Caused by Mutations in Nuclear Genes

Gene Therapy represents the most straightforward “precision medicine” approach for many MDs, although several issues must be considered for its successful use.

Most MDs are multi-systemic syndromes, i.e., they affect several organs; therefore, at least in theory, a widespread, if not ubiquitous, gene expression of the vector carrying the therapeutic transgene should be required to achieve a significant recovery of the patient’s condition. However, it has been demonstrated that single-organ gene therapy could successfully ameliorate the phenotype of MDs at least in two different conditions: the tissue-specific clinical presentation of mitochondrial dysfunction (e.g., LHON, a liver-specific mtDNA depletion syndrome due to mutations in MPV17) and multi-organ involvement due to the systemic accumulation of toxic compounds (e.g., ethylmalonic encephalopathy, mitochondrial neurogastrointestinal encephalomyopathy). These aspects are extensively discussed in Section 3.2, Section 3.3 and Section 4.1.

Among other issues, the high risk of an immune response against the delivery vector or the therapeutic cargo requires practical consideration on how to achieve such a broad expression pattern.

First, high doses of vectors are required to achieve sustained and widespread systemic effects, with related issues regarding toxicity and immunogenicity. Large batches of vectors need to be produced at a high yield, which considerably increases the manufacturing costs and, eventually, the cost of the treatment. Second, a viral particle with the correct tropism must be chosen based on the target tissue. In most cases, systematic delivery across multiple tissues is required. Third, since the CNS is commonly affected in mitochondrial diseases, the viral particle must be able to cross the BBB. Here we review the preclinical and clinical applications of gene therapy to restore the functionality of mutated genes causing MD.

### 3.1. Mitochondrial Myopathy (MM) and Cardiomyopathy

As a paradigmatic example of mitochondrial myopathy, we will discuss gene therapy approaches available for *ANT1*. *ANT1* encodes the adenine nucleotide translocator, an integral inner mitochondrial membrane (IMM) protein operating as electrogenic pumps that export ATP in exchange for cytosolic ADP [81]. Mutations in *ANT1* lead to MM associated with ragged red muscle fibers and PEO triggered by the paralysis of the extraocular eye muscles [81].

One of the earliest preclinical gene therapy studies applied to a mitochondrial myopathy (MM) was performed using the ssAAV2 vector carrying the murine *Ant1* cDNA administered intramuscularly (1 × 10^9^ i.u.) to the *Ant1* knockout (KO) mouse model. The *Ant1* KO mouse manifested severe exercise intolerance with metabolic acidosis. The histological and ultrastructural analysis revealed the presence of ragged red muscle fibers with mitochondrial defective coupled respiration, while examination of the heart revealed cardiac hypertrophy with mitochondrial proliferation. AAV2-*Ant1* transduction resulted in long-term stable expression in muscle precursor cells and differentiated muscle fibers. The transgenic ANT1 protein was targeted to the IMM and formed a functional ADP/ATP carrier, resulting in a 5–30% increase in ANT1 protein, a 25–45% increase in ATP production, and a reversion of the histopathological changes associated with the MM [82].

### 3.2. Metabolic Disorder Caused by the Accumulation of Toxic Compounds

We discuss the gene therapy approaches available for ethylmalonic encephalopathy caused by mutations on *ETHE1* as an emblematic example of mitochondrial encephalopathy caused by the toxic accumulation of sulfide. *ETHE1* is a nuclear gene encoding sulfur dioxygenase (SDO), which takes part in the mitochondrial pathway that converts sulfide into harmless sulfate. Recessive mutations in *ETHE1* lead to ethylmalonic encephalopathy (EE), a fatal mitochondrial disease characterized by the accumulation of hydrogen sulfide (H_2_S) and ethylmalonic acid (EMA) [83]. At higher concentrations, H_2_S acts as a toxic compound that inhibits several enzymes, such as cytochrome c oxidase (COX) [84] and short-chain acyl-CoA dehydrogenase [85], leading to the progressive accumulation of necrotic and hemorrhagic brain lesions [86], chronic hemorrhagic diarrhea, vascular petechial purpura, and orthostatic acrocyanosis.

The *Ethe1 KO* mouse shows growth arrest from postnatal day 15 and reduced motor activity with premature death between the fifth and sixth week after birth. Moreover, it partially recapitulates patients’ biochemical alterations, with markedly low COX activity in the muscle, brain, and jejunum but normal activity in the liver. The clearance of circulating H_2_S by expressing the missing *ETHE1* gene in a filtering organ such as the liver could decrease the levels of H_2_S, thus acting as a detoxifying treatment.

A single systemic injection of 4 × 10^13^ viral genomes (vg)/kg of an ssAAV2/8 vector expressing the human *hETHE1* cDNA under the liver-specific thyroxine-binding globulin (TBG) promoter in three weeks early symptomatic *Ethe1* KO mice resulted in a marked amelioration of the phenotype and a robust prolongation of the mouse lifespan. This remarkable clinical result was associated with the partial or complete correction of the disease’s main metabolic and biochemical indexes, including the EMA and thiosulfate levels in plasma and the COX activity in tissues [87] Living-donor orthotopic liver transplantation also resulted in an effective option to treat EE since the transplanted organ substituted the deficient ETHE1 enzyme and cleared the circulating toxic H_2_S [88,89,90]. These results proved the efficacy and safety of AAV2/8-mediated liver gene therapy for EE and similar conditions caused by the accumulation of toxic compounds in body fluids and tissues.

### 3.3. Mitochondrial DNA Depletion Syndrome

Mitochondrial DNA depletion syndrome (MDS) is an autosomal recessive condition characterized by variable organ involvement with decreased mtDNA copy number and reduced enzymatic mitochondrial respiratory chain activities in the affected tissues. The depletion of mtDNA in humans has been associated with mutations in nine nuclear genes, six of which (*TYMP*, *TK2*; *DGUOK*; *SUCLA2*, *SUCLG1*, and RRM2B) are involved in the homeostasis of the mitochondrial nucleotide pool [91]. In addition, mtDNA depletion can be caused by mutations in *POLG*, encoding the catalytic subunit of mitochondrial polymerase γ; *PEO1*, encoding a mitochondrial T4-phage-like helicase (twinkle) [92]; and *MPV17*, encoding an inner mitochondrial membrane protein of unknown function [93]. Several gene therapy approaches have been explored to restore the physiological activity of TYMP, TK2, and MPV17 [94].

#### 3.3.1. *TYMP*

Mitochondrial neurogastrointestinal encephalomyopathy (MNGIE) is caused by autosomal recessive mutations in the nuclear gene TYMP, which encodes the cytosolic enzyme thymidine phosphorylase (TP). The lack of TP activity leads to the systemic accumulation of TP substrates, the nucleosides deoxythymidine (dT) and deoxyuridine (dU), which are precursors of the deoxyribonucleoside triphosphates (dNTPs) used for DNA synthesis [83].

This nucleoside perturbation alters the mitochondrial dNTP pool, leading to dysfunctional mtDNA replication, mtDNA depletion, multiple deletions, and point mutations in specific tissues. MNGIE patients develop gastrointestinal dysmotility, progressive external ophthalmoplegia, peripheral neuropathy, diffuse leukoencephalopathy on brain magnetic resonance imaging, and mitochondrial dysfunction. The *Tymp/Upp1* mouse was used during the last decade to implement different preclinical gene therapy strategies. The *Tymp/Upp1* double KO mouse model has reduced TP activity, increased dT and dU concentrations, reduced activities of complex I (CI) and complex IV (CIV), and late-onset vacuoles in the cerebral and cerebellar white matter without demyelination or axonal loss. One of the first strategies developed for restoring TP and reverting dT and dU overload to normal levels was the lentiviral-mediated hematopoietic ex vivo gene therapy [95]. Immunoselected hematopoietic-lineage-negative (Lin−) cells from dKO mice were transduced with a lentiviral vector containing the TYMP cDNA (p305-TP), holding the human phosphoglycerate kinase (hPGK) promoter, and including the EGFP-encoding sequence as a marker gene. The transduced cells were infused into partially myeloablated syngenic dKO mice. One month after transplantation, the level of gene marking in the peripheral blood (PB) of TP-transplanted mice ranged between 2.0 and 10.5%, with a mean copy number per transduced cell ranging from 0.5 to 1.5. High levels of TP activity and a parallel reduction in nucleoside concentrations were detected in the peripheral blood of the transplanted mice.

However, a long-term follow-up revealed a reduced survival rate of treated mice due to the transplantation procedure, which included total body irradiation of recipient animals before progenitor cell infusion [96].

An alternative approach based on an AAV vector targeting the liver also prevented biochemical imbalances in mice. IV administration of an ssAAV2/8 expressing the human *hTYMP1* under the control of the TBG promoter at doses as low as 2 × 10^11^ vg/kg led to a permanent reduction in systemic dT and dU concentrations to normal values in about 50% of treated mice [97]. Higher doses (10^12^ and 2 × 10^12^ vg/kg) resulted in faster reductions in plasma nucleoside concentrations, which reached physiological levels in 1 week and persisted over the entire monitoring time (88 weeks) [98]. Although significant, these studies are limited because the *Tymp/Upp1* mouse does not develop relevant clinical abnormalities. To overcome this issue, Hirano and collaborators adopted a strategy to exacerbate the clinical phenotype, treating the *Tymp/Upp1* KO mice with both dT and dU in drinking water from weaning [99]. Compared to untreated animals, the treated mice showed reduced survival, body weight, and muscle strength. Furthermore, the treated mutants developed an enhanced leukoencephalopathy, and the small intestine showed a reduction in smooth muscle cells and increased fibrosis. Moreover, mtDNA depletion was detected in the brain and the small intestine, and a deoxyribonucleoside triphosphate imbalance was observed in the brain.

In one elegant study, Marti and collaborators used this deoxynucleoside-stressed model to implement a new gene therapy protocol. They tested the expression of the AAV2/8-delivered *TYMP* transgene with different promoter sequences, i.e., TBG, PGK, hybrid liver-specific promoter (HLP), alpha-1-antitrypsin (AAT), and different DNA configurations, such as single-stranded or self-complementary [100]. Then, 8- to 11-week-old KO mice received therapeutic vectors via a single IV injection at different doses (5 × 10^11^, 10^12^, 2 × 10^12^, and 10^13^ of AAV-TBG; and AAV-AAT or AAV-HLP, both at doses of 2 × 10^12^ and 10^13^ vector genomes per kg, vg/kg). All treatments restored liver TP activity and normalized nucleoside homeostasis in mice. The *Tymp/Upp1* mouse was recently used to implement a liver-directed genome editing approach [101]. In this study, the human *TYMP* transgene was inserted into introns of the *Tymp* and *Alb* loci of hepatocytes by the coordinated delivery and activity of CRISPR/Cas9 and a *TYMP* cDNA. The CRISPR/Cas9 complex was delivered either in an AAV2/8 viral vector or as mRNA using polymeric or lipid nanoparticles. The AAV2/8 virus was also used for packaging the *TYMP* cDNA. However, lipid nanoparticles carrying the CRISPR/Cas9 mRNAs retrieved the best in vivo results. Treated mice had a reliable long-term (1 year) reduction in plasma nucleoside (thymidine and deoxyuridine) concentrations, which correlated with the expression of the TYMP mRNA and the functional enzyme in the liver. These results suggest that gene editing could represent a valid alternative to classical AAV gene augmentation to circumvent this issue.

#### 3.3.2. *TK2*

*TK2* is a nuclear gene encoding for a deoxyribonucleoside kinase with mitochondrial localization that is involved in the salvage pathways for pyrimidine. TK2 phosphorylates both deoxycytidine (dC) and dT to generate deoxycytidine monophosphate (dCMP) and deoxythymidine monophosphate (dTMP), which are subsequently phosphorylated to dCTP and dTTP, which are essential for the replication and maintenance of mtDNA. Recessive mutations in the human *TK2* gene cause a fatal early-onset myopathic form of the mitochondrial depletion/multiple deletions syndrome [102].

*Tk2* knock-in mice holding an H126N (c.378–379CG > AA) mutation, homologous to the human H121N *TK2* mutation, appeared normal at birth. However, after post-natal day 10, the homozygous mutant animals showed growth deceleration, reduced spontaneous activity, generalized coarse tremor, severely impaired gait, and rapidly developed weakness, causing death before 15 days. Hirano’s group used this model to set up three different preclinical gene therapy protocols [103].

The first protocol observed an ssAAV9-TK2 single-dose retro-orbital injection at d1 efficiently rescue Tk2 activity in all the tissues except the kidneys, delay disease onset, improv disease course, and increase lifespan (from a median of 16 days to 88.5 days).

The second strategy observed a sequential treatment with ssAAV9 first (d1) followed by ssAAV2 on d29. This combined treatment reduced the viral dose while further prolonging the lifespan (to a median of 119 days). The third strategy (ssAAV9 + ssAAV2 combined with dC and dT supplementation, 520 mg/kg/day each) dramatically improved the mtDNA copy numbers in the liver and kidneys, animal growth, and lifespan (to a median of 481 days).

#### 3.3.3. *MPV17*

*MPV17* is a nuclear gene encoding for a mitochondrial inner membrane protein involved in mitochondrial deoxynucleotide homeostasis and the maintenance of mtDNA [93]. Mutations in human *MPV17* cause a hepatocerebral form of MDS hallmarked by early-onset severe hypoglycemic crises followed by liver cirrhosis and failure leading to premature death [104]. Patients who survive liver failure develop progressive peripheral neuropathy and cerebellar degeneration [105].

The KO mouse for *Mpv17* is characterized by severe early-onset mtDNA depletion in the liver, a degeneration of the inner ear structures, leading to hearing loss, and late-onset, fatal kidney dysfunction with focal segmental glomerulosclerosis that causes proteinuria.

Although the molecular and biochemical features in the liver of *Mpv17 KO* mice closely resemble human alterations, this model does not develop hepatic dysfunction and neuropathological abnormalities [106]. However, when fed a high-fat ketogenic diet (KD), *Mpv17 KO* mice rapidly develop liver cirrhosis and failure. 

In 2014, Bottani and collaborators implemented a preclinical gene therapy protocol for MPV17-MDS based on an ssAAV2/8 viral vector expressing the *hMPV17* cDNA under the control of the liver-specific TBG promoter that was systemically administered by retroorbital injection at a dose of 4 × 10^12^ vg/kg in 2-month-old *Mpv17 KO* and control mice. A blood sample analysis before the AAV treatment revealed higher levels of alanine aminotransferase and aspartate aminotransferase enzymes, two markers of hepatocyte leakage. These markers were fully normalized three weeks after AAV injection. Moreover, this treatment effectively prevented KD-induced liver degeneration and restored mtDNA depletion [94].

### 3.4. Leigh Syndrome

Leigh syndrome (LS) is a neurometabolic MD that affects 1 in 36,000 newborns and causes lactic acidosis and symmetric lesions in the CNS, leading to intellectual disability and muscle weakness, with a peak of mortality before three years of age. Mutations in more than 75 genes of nuclear or mtDNA that commonly affect CI and CIV of the mitochondrial respiratory chain can cause LS [107]. The following chapters will discuss the gene therapy approaches recently exploited in KO mouse models for *Ndufs4* and *Surf1*.

#### 3.4.1. *NDUFS4*

NDUFS4 is a non-enzymatic, 18 kDa nuclear-encoded CI subunit. Mutations in the *NDUFS4* gene are a frequent cause of LS [108]. Common symptoms include psychomotor arrest or regression, hypotonia, dystonia, ataxia, ophthalmoplegia, lethargy, apneic spells, and respiratory failure with elevated lactate levels in the blood and cerebrospinal fluid [109].

The constitutive *Ndufs4* KO mouse model appeared healthy at birth, but starting from 40 days of age, it develops progressive encephalopathy, growth retardation, ataxia, hypotonia, and lethargy, ultimately leading to death at ~7 weeks. Notably, CI activity was reduced, recapitulating the pathophysiology observed in LS patients [110].

Different research groups have used this model to develop a gene therapy strategy for LS during the last decade. Di Meo and colleagues delivered the human wild-type *NDUFS4* cDNA under the control of the cytomegalovirus (CMV) promoter via an ssAAV2/9 vector. The vector was administered (2 × 10^12^ vg/mouse) IV by retroorbital injection at postnatal day 21. High concentrations of the hNDUFS4 protein were found in the skeletal muscle, heart, and liver; however, no significant increase in protein level was detected in the brain. Although hNDUFS4 was able to fully restore the CI assembly in *Ndufs4* KO liver mitochondria and rescue the CI spectrophotometric activity in the viscera, no noticeable improvement in the clinical phenotype was observed. Similar results were obtained in newborn mice injected systematically through the temporal vein. A slight increase in body weight and a significant improvement in motor coordination, without lifespan extension, were obtained in newborn mice injected intracerebroventricularly (ICV) with a higher dose (3 × 10^11^ vg/mouse). Importantly, an imaging investigation of injected brains revealed that most of the transduced cells belonged to the glia.

Finally, double administration of AAV2/9-hNDUFS4 by both IV and ICV administration caused a remarkable improvement in the clinical symptoms, such as an amelioration of motor coordination, a gain of body weight, and a highly significant prolongation of the lifespan (82 days) compared with untreated KO mice [111].

Based on these results, the group implemented the protocol using the PHP.B serotype, characterized by an efficient capacity to cross the BBB when administered IV. A single IV tail injection (10^12^ vg/mouse) of ssAAV.PHP.B-hNDUFS4 in adult (postnatal day 26–28) *Ndufs4* KO mice transduced both neurons and glial cells effectively throughout the brain, leading to the restoration of NDUFS4 protein expression in the brain and visceral organs, normalizing CI activity and supercomplex formation. Treated animals a showed recovery of body weight and improved coordination performance with a delayed onset of neurodegeneration and a prolongation of the lifespan of up to 100 days. Curiously, the administration of ssAAV.PHP.B-hNDUFS4 to newborn mice (postnatal day 1, temporal vein injection, 10^12^ vg/mouse) was ineffective. Ex vivo analyses showed that injected pups and adults displayed similar viral genome copy numbers in the extracerebral tissues, with a higher transduction of skeletal muscle. However, the copy number in the pup brains was lower than in adults, suggesting that the AAV-PHP.B crossed the BBB much less effectively in pups than in adults. Accordingly, neonatally treated mice developed ataxia and died at 40 days post-administration, similar to untreated mice [112].

Decressac and colleagues obtained similar results, which showed how the IV delivery (10^12^ vg/mouse) of an ssAAV.PHP.B expressing *Ndufs4* under control of the chicken-β-actin CBA promoter to 1-month-old KO mice was able to restore CI activity in several organs, including the CNS. This gene replacement strategy extended lifespan (+250 days), rescued metabolic parameters, provided behavioral improvement, and corrected the pathological phenotype in the brain, retina, and heart [113].

#### 3.4.2. *SURF1*

*SURF1* is a nuclear gene encoding for a mitochondrial inner membrane protein involved in the assembly of CIV. *SURF1* mutations lead to defective COX assembly and severe COX deficiency, representing one major cause of LS [114]. The constitutive *Surf1* KO murine model develops mild COX deficiency and a slight elevation of blood lactate but fails to recapitulate the human clinical signs while displaying a surprising increase in longevity and enhanced memory [115]. Despite the absence of a relevant neurological phenotype, this model was recently used to evaluate gene therapy.

An scAAV9 vector carrying *hSURF1* cDNA under the control of the hybrid chicken-β-actin/CMV enhancer (CAG) promoter was delivered through an intrathecal injection (8 × 10^11^ vg/mouse) to one-month-old KO mice. The treatment effectively increased the SURF1 mRNA expression in multiple relevant organs, including the brain and spinal cord, leading to a partial recovery of the COX activity in all tissues as well as the abnormal lactate acidosis induced by exhaustive exercise [116].

### 3.5. Friedrich Ataxia

Friedreich ataxia (FRDA) is a rare neurodegenerative disease that is characterized by spinocerebellar and sensory ataxia and is associated with hypertrophic cardiomyopathy and diabetes [117] FRDA is mainly caused by a homozygous n(GAA) expansion within the first intron of the frataxin gene (*FXN*), an essential mitochondrial protein involved in the biosynthesis of iron–sulfur (Fe-S) clusters. This triplet expansion leads to the heterochromatinization of the locus, with a consequent reduction in *FXN* transcription. A frataxin deficiency leads to impaired Fe-S biogenesis, the impairment of Fe-S enzymes, mitochondrial dysfunction, iron metabolism dysregulation, and eventually cellular dysfunction and death [118].

Several disease models have been developed over the last few years, but many of these failed in recapitulating the clinical aspects of FRDA. Please refer to this manuscript [119] for a detailed summary of the characteristics of the main FXN mouse models. Nevertheless, these models helped implement gene therapy protocols. Puccio et al. treated the 7-week-old conditional mouse model (Mck-Cre-FxnL3/L) with an ssAAVrh10-CAG-hFXN-HA vector at a dose of 5.4 × 10^13^ vg/kg by retro-orbital injection. The treatment was effective in preventing or reversing cardiomyopathy and mitochondrial dysfunction, whether injected before or after the onset of the pathology [120].

In a later study, the same group generated a *Pvalb-Cre* conditional KO model that develops ganglionopathy with sensory axonopathy and cerebellar ataxia. A single IV injection of ssAAV9-CAG-FXN-HA at a 5 × 10^13^ vg/kg dose was performed in 3.5-week-old early symptomatic *Pvalb* KO mice. All performed behavioral tests showed a significant improvement in motor coordination compared to untreated mice. Moreover, to determine the therapeutic potential of the treatment at a later post-symptomatic stage, IV administration of ssAAV9-CAG-FXN-HA at a dose of 5 × 10^13^ vg/kg simultaneously with the intracerebral delivery of ssAAVrh.10-CAG-FXN-HA (1 × 10^10^ vg/kg) was performed post-symptomatically at 7.5 weeks of age. The treatment reversed the fine peripheral coordination and neurological features within the first few days after treatment [120]. Despite these promising results, it is essential to carefully consider that the dose of vector administered following the dual injections was very high and might not be applicable in a clinical setting. A recent study demonstrated the safety of FXN cardiac overexpression up to nine times the physiological level but significant cardiac toxicity above twenty times. The toxicity seems to be caused by mitochondria respiratory chain and ultrastructure dysfunction, which leads to cardiomyocyte subcellular disorganization, cell death, and fibrosis [121]. Therefore, expressing high concentrations of FXN requires a careful evaluation of potential cytotoxicity.

## 4. Gene Therapy for mtDNA-Associated Disorders

### 4.1. Allotopic Expression

Allotopic gene expression is a strategy to overcome mtDNA mutations by re-expressing the missing mtDNA-encoded protein from the nucleic genome [122]. In this case, physiologically mtDNA-encoded proteins are imported into the mitochondria via an MTS following the nuclear expression of an engineered nuclear version of a mitochondrial gene.

#### Leber’s Hereditary Optic Neuropathy

The first attempt of this strategy was carried out for Leber’s hereditary optic neuropathy (LHON), a maternally inherited disease associated with homoplasmic mtDNA mutations and the most frequent mitochondrial disease, which causes blindness in young people, particularly males. The prevalence of LHON has been estimated at between 1 in 30,000 and 1 in 50,000 in northern Europe. The pathology is characterized by the selective degeneration of retinal ganglion cells (RGCs) and optic nerve atrophy, leading to central vision loss. Over 95% of all cases are due to pathogenic mutations in one of three mitochondrial genes that encode CI subunits of the respiratory chain: *ND1*-G3460A, *ND4*-G11778A, or *ND6*-T14484C. These mutations have the double effect of chronically lowering ATP synthesis and increasing oxidative stress [123].

Pharmacological treatments that reverse the visual loss of LHON patients have yet to be developed; however, gene therapy has obtained promising results. The eye is an ideal candidate for local gene therapy due to its relatively small size and limited local immune and inflammatory responses (known as ocular immune privilege) [124]. Moreover, the affected RGCs are located on the inner surface of the retina, an easily accessible site via standard intravitreal injection. For these reasons, multiple trials are currently investigating the treatment of LHON using an AAV2 vector designed to promote an allotopic expression of ND4. The first LHON animal model was created by introducing the human *ND4* gene harboring the G11778A mutation to rat eyes by in vivo electroporation. The treatment induced the degeneration of RGCs, followed by a decline in visual performance. A subsequent electroporation with the wild-type *ND4* sequence prevented RGC loss and the impairment of visual function [125] In a subsequent study, Guy and colleagues demonstrated that an MTS-targeted AAV carrying the mitochondrial gene encoding the human *ND4* prevented the optic atrophy induced by the mutant R340H *ND4* [126].

Currently, different research groups are investigating the safety and the efficacy of the intravitreal delivery of AAV2 vectors to promote the allotopic expression of the human ND4 (NCT03153293, NCT03293524, and the completed study NTC01267422. For detailed information about the clinical trials, visit https://www.clinicaltrials.gov/ct2/results?cond=LHON&term=ND4&cntry=&state=&city=&dist= (accessed on 4 April 2022).

In addition, two randomized, double-masked, sham-controlled phase 3 clinical trials (REVERSE, NCT02652780, and RESCUE, NCT02652767) and their long-term follow-up study (RESTORE, NCT03406104) showed a bilateral improvement in best-corrected visual acuity in patients who were unilaterally treated [127]. Similar results were obtained in two other trials [128,129].

A study performed on a non-human primate demonstrated that the transfer of viral vector DNA from the injected eye to the noninjected eye provides insights into the possible mechanisms of this apparent contralateral therapeutic effect [130]. Despite the reported beneficial effect, results from the ongoing trial (NCT02064569) revealed, as side effects, an inflammatory response: thirteen patients suffered intraocular inflammation as a consequence of rAAV2/2-ND4 administration. All cohorts treated with different doses of the vectors experienced mild anterior chamber inflammation and vitritis, and all cases were responsive to treatment [131]. It is important to note that allotopic gene therapy cannot represent a permanent cure because the actual cause of the disease, such as the G11778A point mutation in the mitochondrial genome, is still present. The mtDNA gene editing represents a promising resolutive strategy that directly corrects or removes the mtDNA point mutation.

### 4.2. Mitochondrial Delivery of Nucleic Acids

Most of the current gene therapy approaches focus on replacing or correcting a mutant sequence using a wild-type copy of the altered gene. However, the current inefficiency in delivering nucleic acids to mammalian mitochondria and the high mtDNA copy number represent a teething issue in developing efficient gene therapies for mtDNA disorders. Therefore, a more targeted approach aiming to deliver the therapeutic cargo directly to the affected mitochondria could represent a more feasible approach to treating many MDs [132].

The first attempts to deliver nucleic acid into the mitochondrion relied on liposome-based nanocarriers. The use of dequalinium-based liposome-like vesicles [133], which represent the prototype for all the mitochondrial-targeted vesicular nanocarrier systems, has been successfully explored to deliver DNA and low-molecular-weight molecules to mitochondria within living mammalian cells [134]. Similarly, Mito-Porter is a liposome-based carrier that is able to release macromolecular cargos into the mitochondrial matrix either in isolated mitochondria or in living cells through membrane fusion [135]. This approach was used in a proof-of-principle preclinical study. Positive results were reported following the wild-type mitochondrial RNA delivery in the patient’s cells carrying the heteroplasmic tRNA^Phe^ G625A [136] and the ND3 T10158C mutations [137].

A further intriguing strategy exploits the RNA import complex [138], a multi-subunit protein complex found in the mitochondria of the kinetoplastid protozoon *Leishmania tropica*, which can import nucleus-encoded tRNAs into mitochondria [139]. The RNA import complex was exploited to import endogenous cytosolic tRNAs, including tRNA^Lys^, and restored mitochondrial function in wild-type, MERFF, and KSS cybrids [138]. Finally, a further attempt to efficiently deliver DNA material to mitochondria relied on the bioengineering of an ssAAV2 vector. An MTS was fused to the VP2 protein, forming the modified viral capsid MTS-AAV. Although successful gene delivery to the mitochondria of LHON mice was observed following intravitreal injections, the mechanisms by which the vectors enter the organelle and how the encapsulated DNA is released into mitochondria still remain unclear [140].

### 4.3. mtDNA Heteroplasmy Manipulation

Together with the nature of the mtDNA defect (i.e., point mutation versus large deletions), the percentage of heteroplasmy is the predominant factor determining the clinical and biochemical manifestations of MDs. In particular, the mutant-to-wild-type ratio of mtDNA molecules has to exceed a specific limit to impact OXPHOS and cause a mitochondrial phenotype, an event known as the threshold effect. This threshold varies between 60% and 90% of mutant mtDNA, depending on several factors, including the specific mutations and the affected organ and the individual variability [141]. In this context, moving the mtDNA heteroplasmy below the pathogenic threshold offers a rational approach to cure MDs. Therefore, strategies to manipulate mtDNA have been developed to eliminate or reduce the amount of mutated mtDNA to favor the wild-type mitochondrial genomes and ameliorate the severity of the disease, a methodology referred to as heteroplasmy shifting. Taking advantage of the rapid degradation of mtDNA induced by double-strand breaks (DSBs), this strategy exploits the site-specific breakage of mutant mtDNA molecules, leading to its elimination with concomitant selection and repopulation by the wild-type form, ultimately restoring the normal mitochondrial functions. Recently, several approaches have been developed based on the specific targeting of recombinant restriction endonucleases (mitoREs), zinc-finger nucleases (ZFNs), or transcription activator-like effector nucleases (TALENs) to mitochondria. Starting from the pioneering proof-of-principle work by Srivastava and Moraes [142], the first attempt to eliminate human disease-associated mutated mtDNA molecules was carried out in 2002 through the transient expression of a recombinant *SmaI*-mitoRE into cybrids carrying the pathogenic m.8993T > G mutation, associated with NARP and MILS. The patient’s derived fibroblasts were repeatedly transfected with the construct containing the gene encoding the restriction endonuclease *SmaI* and selected for wild-type mtDNA expression [143]. The specific degradation of mutated mtDNA resulted in repopulation by the wild-type genome and the restoration of some of the mitochondrial biochemical features to physiological levels. The efficacy of mitoREs has also been demonstrated in vivo using AAV or adenoviral viral vectors injected locally or systemically in heteroplasmic mice [144,145,146,147]. However, although mitoREs have proven effective in changing mtDNA heteroplasmy both ex vivo and in vivo, their application is limited since very few clinically relevant heteroplasmic mtDNA mutations create endonuclease restriction sites for naturally occurring enzymes. To overcome this limitation, different groups have recently explored the use of programmable endonucleases with modular DNA recognition domains, which can be designed to recognize specific sequences. ZFNs [148] and TALENs [149] are the most promising systems, sharing a common basic structure composed of the catalytic subunit of the type II endonuclease *FokI* coupled to a sequence-specific modular DNA-binding domain. In ZFNs, each individual zinc-finger domain recognizes three nucleotides, while for TALENs, each TALE domain recognizes one nucleotide. This means that appropriate arrangements of zinc-finger or TALE modules allow targeting virtually any DNA sequence. Both systems can easily enter mitochondria by adding an MTS at the N-terminus. Moreover, since *FokI* works as a dimer, both enzyme systems require the design of pairs of monomers that bind the region of interest tail–tail nearby, allowing the dimerization of *FokI* domains and double-strand cleavage. Mitochondrially targeted ZFN (mtZFN) technology was first used on cybrid cells to target pathogenic genomes harboring the m.8993T > G mutation or the m.8483_13459del4977 large deletion [150,151,152]. Likewise, mitochondria-targeted TALENs (mitoTALENs) eliminated mutant mtDNA in cybrids carrying the heteroplasmic m.8483_13459del4977 large deletion or the LHON-associated m.14459G > A mutation in the MT-ND6 gene [153]. AAV vectors were recently used to deliver ZFNs and mtZFNs in vivo to eliminate mutated mtDNA selectively. In a mouse model of heteroplasmic mitochondrial disease bearing the point mutation m.5024C > T in MT-tRNA^ALA^ [154], both systems were able to reduce the mtDNA heteroplasmy with the concomitant rescue of the molecular and biochemical phenotypes [155,156]. However, both systems’ heterodimeric natures and large sizes could represent a disadvantage for viral-vector-based gene therapy approaches. More recently, an adaptation of the novel gene-editing platform based on the *Chlamydomonas reinhardtii* chloroplast-genome-derived *I-CreI* meganuclease (mitoARCUS), delivered via an AAV9 vector, has been successfully used in vivo to eliminate the mutated mtDNA in skeletal muscle specifically and the heart and liver of the m.5024C > T heteroplasmic mouse model [157].

### 4.4. mtDNA Editing

While holding a promising potential, heteroplasmy shifting approaches cannot be used to target homoplasmic mutations and cannot introduce specific nucleotide changes in mtDNA. These approaches could also be harmful if used in the presence of a high load of heteroplasmic mutations since the sudden elimination of the majority of the mtDNA could be detrimental to sensitive cellular populations. The possibility of developing a DNA-editing-based gene therapy approach to stably revert a specific mitochondrial mutation represents one of the milestones chased by the whole scientific community. However, the difficulty of targeting RNAs to mitochondria has hindered the development of such tools, including the guide RNA required for CRISPR-Cas9 technology [158]. Recently, a precision base editing approach using a cytidine deaminase efficiently converted C to T (or G to A) in the mtDNA of cultured cells [159]. This groundbreaking result was obtained thanks to the discovery of DddAtox, a bacterial cytidine deaminase isolated from *Burkholderia cenocepacia* that can catalyze the deamination of cytidines on dsDNA. Split into two inactive parts until simultaneously carried on the target sequence, DddAtox was reconstituted on the target mtDNA site by an adjacently bound mitochondrially targeted TALE array. The addition of a uracil glycosylase inhibitor, which prevents the uracil excision during cytidine deamination, generated the RNA-free DddA-derived cytosine base editors (DdCBEs), which permit guided CG-to-TA changes in mtDNA without the need for DSBs. This technology was immediately exploited to edit the mtDNA in mice [160] and zebrafish [161] through DdCBE mRNA delivery to embryos. Minczuk and collaborators successfully applied an in vivo proof-of-concept editing approach based on AAV-delivered DdCBE. They demonstrated the possibility of editing a specific mtDNA nucleotide in the heart of neonatal and adult mice [162], thus providing, for the first time, its potential translation to human somatic gene correction therapies to treat primary mitochondrial disease phenotypes. However, the identification of low-frequency off-target editing in the nuclear genome of mouse embryos demonstrates the solid need to optimize DdCBE for specific base editing on mtDNA, especially before being used for treating mitochondrial diseases [163]. Despite the scientific community’s enthusiasm, this technology is mostly limited to cytosine editing in the 5′-TC context. As a result, DdCBEs can only fix 9 (=10%) of 90 proven deleterious point mutations.

Cho and colleagues [164] recently presented TALE-linked deaminases (TALEDs), which are made up of custom-designed TALE DNA-binding arrays, a catalytically impaired full-length DddA variant or split DddA from *Burkholderia cenocepacia*, and an engineered deoxyadenosine deaminase derived from the *E. Coli* TadA protein, all of which induce targeted A-to-G editing in human cells. The custom-designed TALEDs catalyzed A-to-G conversions at a total of 17 target sites in diverse mitochondrial genes, with editing frequencies as high as 49%. In the short term, TALEDs could be used for creating disease models by inducing mtDNA mutations in cell lines and animals, which is an important step in mitochondrial medicine. In the long run, TALEDs could be used to correct 39 (=43%) out of the 90 pathogenic mutations, including those causing LHON, MELAS, and LS.

More efficient and selective TALEDs could pave the way for the correction of disease-causing mtDNA abnormalities in embryos, fetuses, newborns, and adult patients, ushering in a new era of mitochondrial gene therapy.

## 5. Future Perspective

### 5.1. In Utero Fetal Gene Therapy

Advances in prenatal genetic diagnosis and progress in fetal surgery have led to the development of interventional protocols for the in utero treatment of different fetal disorders, such as congenital diaphragmatic hernia, myelomeningocele, pulmonary sequestration, hydrothorax, urinary tract obstruction, and so on [165].

With the implementation of sophisticated imaging and endoscopic techniques, in utero fetal gene therapy (IUFGT) is becoming a realistic option for congenital early-onset conditions where the aim is now to improve quality of life rather than extend survival.

IUFGT plans to deliver genes to cells and tissues early in prenatal life, offering the potential of prophylaxis, allowing the correction of a genetic defect before early irreparable tissue damage has occurred and, overall, improving postnatal clinical outcomes. In contrast to postnatal gene therapy, the prenatal application may offer several advantages: (i) the small fetal size allows for a higher vector-to-target-cell ratio to be achieved; (ii) the tolerogenic fetal immune system could limit anti-vector immune responses, often acquired in postnatal life, and could facilitate postnatal repeat vector administration if needed; (iii) the presence of highly proliferative and accessible stem/progenitor cells in multiple organs; (iv) the BBB is most permeable during early developmental stages, increasing brain transduction; and (v) the ability to treat disorders in which irreversible pathological metabolic and molecular changes begin before birth [166].

From a technical standpoint, potential genetic therapeutic agents can be delivered to the fetus through transabdominal intrauterine ultrasound-guided injection in the umbilical cord vessels or direct injection into the fetal organs. In addition, the surgery skills and techniques required for IUFGT are similar to well-established methods used for umbilical-vein blood transfusion in human fetal medicine.

**Figure 4:**
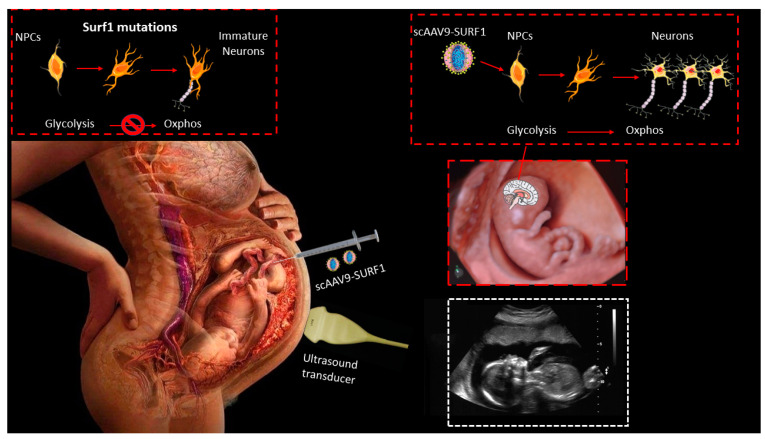
Schematic representation of IUFGT to restore correct neurodevelopment in Surf1 LS-affected fetus. The therapeutic gene can be delivered to the fetus through transabdominal intrauterine ultrasound-guided injection of AAV9-Surf1 in the fetal umbilical cord vessels. Figure 4 was modified from SMART (Servier Medical Art) and licensed under a Creative Common Attribution 3.0 Generic License. http://smart.servier.com/ (accessed on 16 April 2022 ).

Prenatal gene therapy could be beneficial for early-onset mitochondrial disease in which mitochondrial dysfunction may affect the correct development before birth, as in GRACILE syndrome, which causes intrauterine growth retardation and leads to a fatal lactic acidosis, often accompanied by nonspecific aminoaciduria, cholestasis, iron overload, and liver dysfunction in newborns [167]. Recent findings indicate that *SURF1* mutations cause metabolic impairments in neural progenitor cells, which fail to switch from glycolytic to OXPHOS metabolism, with subsequent aberrant proliferation and insufficient support for neuronal morphogenesis [167,168]. Similarly, we reported a severe neuronal impairment in a *SURF1* KO pig model [169]. Cerebral organoids from LS patients carrying homoplasmic MT-ATP6 mutations also showed defective corticogenesis, suggesting a pre-natal defect [170]. Altogether, these observations indicate that an OXPHOS defect could impair neural stem cell metabolism in the early phase of neuronal development, leading to the onset of neurological phenotypes. Therefore, IUFGT may represent the resolutive therapy for LS, offering the possibility of early intervention before irreversible damage in a temporal window in which undamaged neurons can be harnessed for transgenic protein production, increasing the chances of restoring OXPHOS activity and complete neural differentiation (Figure 4). Recently, the safety and efficacy of IUFGT were tested in a mouse model of neuronopathic Gaucher’s disease [170] using a fetal intracranial injection of an AAV9 vector. The curative gene ameliorated neuronal inflammation and outstandingly increased the overall survival of treated mice. Notably, a neonatal treatment did not obtain the same results as fetal therapy.

After IUFGT, therapeutic viruses, including AAV vectors, may undergo random integration in the fetal genome; however, there have been no reports of germline integration so far. In the future, new technologies with more precise gene editing may reduce IUFGT off-target events.

Despite the promising preliminary results of IUFGT, further experimental evidence in animal models is needed to show a significant improvement in the disease’s pathological markers and clinical progression.

Clinical trials, including human pregnancies, would need to be set up with accurate adverse event tracking and long-term postnatal clinical follow-ups. In addition, there is currently no screening protocol for uncommon genetic disorders in the overall low-risk population. Consequently, most patients currently receiving a prenatal diagnosis of MD have already given birth to a child who was previously affected.

### 5.2. Germline Gene Therapy

Germline gene therapy (GGT) is a type of gene therapy that targets reproductive cells (sperm and eggs) or preimplantation embryos. GGT can potentially correct disease-causing mutations to wild-type variants early in development when the mutation is present in one of few embryonic cells. Furthermore, GGT will prevent the transmission of genetic disease to a child and his/her future generations [171].

Unlike somatic gene therapy, the random integration of a transgene into a nonspecific location within the genome is undesirable for GGT due to safety concerns. The use of episomic AAVs to reduce the insertional mutagenesis risk would be inefficient in GGT due to the dilution the viral particles would undergo during the entire development process.

GGT may employ various genetic manipulation techniques, such as gene replacement and gene editing, to achieve the therapeutic goal. In the context of MDs caused by mutations in nuclear genes, gene editing approaches, including RNA-guided CRISPR–Cas9, may represent a promising strategy for implementing GGT due to their high efficiency in targeting insertion and deletion (indel) mutations at specific loci. Recently, a similar approach was used to disrupt the C–C chemokine receptor type 5 (CCR5) gene, encoding a human cell surface protein implicated in HIV-1 entry, in human embryos that resulted in the birth of twin girls in China. The public response was unanimously negative for several ethical implications [172].

From an ethical point of view, GGT is more acceptable when used in the context of mtDNA mutations. GGT may utilize various genetic manipulation methods, including mtDNA editing (discussed above) or mitochondrial replacement therapy (MRT), proposed as a potential method for preventing the transmission of mutated mtDNA from the mother to the offspring by replacing the mitochondria in the oocytes of carrier women. MRT is commonly performed through spindle transfer, which occurs when the nuclear DNA material is integrated into metaphase chromosomes, generating a meiotic spindle at the mature oocyte stage. The spindle is separated into a karyoplast using microsurgery and then transplanted into the ‘empty’ cytoplasm of an unfertilized donor oocyte that has been enucleated. The rebuilt egg can now be fertilized and put into the patient, as it is now free of mutant mtDNA. Transferring polar entities or pronuclei [173] is a similar strategy. The reconstructed oocyte, now free of mutated mtDNA, can be fertilized and transferred to the patient [174]. Similar strategies involve the transfer of polar bodies or pronuclei [173,175].

### 5.3. RNA-Based Therapy

In 2018, two RNA-based drugs were approved to treat hereditary AATR amyloidosis, with many more RNA therapies in the clinic. However, the potential of RNA delivery for the treatment of MDs has not been fully explored. Although many studies aimed to deliver tRNA synthetase enzymes to stabilize mutant mitochondrial tRNAs, the direct administration of RNA molecules to mitochondria has been challenging. Nonetheless, recent progress in the field makes the direct delivery of RNA an attractive alternative for MDs as a rapid and cost-effective therapeutic option [176]. One of the major controversies revolves around the endogenous import of RNA into mammalian mitochondria. Mitochondrial and nuclear RNAs are structurally different, each characterized by their unique codon usage, making the two divergent and potentially incompatible [158,177]. Although the debate on whether endogenous RNA import into mammalian mitochondria is required for physiological cellular functions is still open, a series of reports have suggested that RNA import is indeed possible in recent years. Correcting defects caused by mutations in mitochondrial tRNA has been the focus of RNA therapy for MDs. RNA therapy usually requires the introduction of exogenous molecules into the cells, with clear hindrance in terms of efficiency and reproducibility. Wang and colleagues [178] used a 20-ribonucleotide sequence derived from the H1 subunit to overcome this issue and successfully directly imported both tRNA and mRNA into human mitochondria. Jo and colleagues [179] reported using the CRISPR/Cas9 system to target mitochondria for mtDNA editing using a modified version of the Cas9 enzyme (mitoCas9) explicitly localized to mitochondria.

Interestingly, the gRNAs were not modified with additional import sequences, indicating that the RNAs were endogenously imported into the mitochondria from the cytosol. These results shed a favourable light on gene editing systems to treat MDs. More recently, Yamada developed a novel gene therapy procedure involving delivering therapeutic RNA molecules to mammalian mitochondria in vitro using fibroblasts derived from LS patients carrying the T10158C mutation in the mtDNA. The wild-type ND3 mRNA was delivered by the MITO-Porter system, increasing mitochondrial respiratory activity [137]. Although these preliminary results are promising, RNA therapies for MDs are still hindered by many technical and biological challenges in delivering RNA molecules into the mitochondria.

A recent communication [180] reported that Burlina and his team, in collaboration with US biotech company Moderna, are developing an mRNA-based therapy to treat methylmalonic acidemia (MMA), a rare metabolic disorder affecting 1 in 100,000 live births. MMA is caused by mutations on the MCM gene that encodes for the mitochondrial enzyme methylmalonyl-CoA mutase, which catalyzes the isomerization of methylmalonyl-CoA to succinyl-CoA. MCM deficiency prevents the body from processing specific amino acids and accumulates methylmalonic acid in the kidneys and the CNS, causing kidney and brain damage, seizures, and coma [181]. According to this report, Moderna created a specific messenger that encodes the correct version of the methylmalonyl-CoA mutase to replace the defective enzyme.

Currently, liver and kidney transplants represent the only option available for the most severe cases of MMA; however, this therapeutic option reduces the acid build-up in almost every tissue but not in the cerebrospinal fluid and thus is not effective for neurological damage. The development of an mRNA-based therapy that can target the liver, kidney, and CNS (i.e., using nanoparticles armed with molecules that bind to specific receptors on these endothelial cells’ surface of the BBB) will allow patients to bypass organ transplantation and the consequent need for chronic immunosuppressive therapies.

## 6. Conclusions

Due to MDs’ high clinical, genetic, and biochemical diversity, the small number of patients, and the frequent lack of acceptable preclinical models, the definition of beneficial clinical outcomes and the development of effective medication is complex.

Despite pharmacological or nutraceutical interventions that apply to a wide range of mitochondrial illnesses and may enhance patients’ daily quality of life, these generalist treatments may not address the core cause of the problem. A precision medicine strategy based on gene therapy that incorporates individual variations in genes, age, sex, stage of the disease, and the tissues impaired for each patient could lead to a definitive cure for mitochondrial disease. However, advancements in viral vector technology now allow more difficult circumstances to be targeted. Genetic therapies are still far from becoming routine for mitochondrial disease due to technical and regulatory issues that make this intervention extremely expensive. However, given recent promising advances in viral vector and gene editing technologies, a greater understanding of the best route of administration and progress in prenatal surgery, in the coming decades we expect considerable progress.

## Data Availability

Not applicable.

## Abbreviations

MDs: mitochondrial diseases; OXPHOS: oxidative phosphorylation; ATP: adenosine triphosphate; mtDNA: mitochondrial DNA; MELAS: mitochondrial encephalomyopathy with lactic acidosis and stroke-like episodes; NARP: neurogenic weakness, ataxia, and retinitis pigmentosa; siRNA: small interfering RNA; LVs: lentiviral vectors; LTRs: long terminal repeats; Ad: Adenoviruses; ITR: inverted terminal repeats; AAVs: Adeno-associated viruses; ORFs: open reading frames; rAAV: Recombinant AAV vectors; scAAV: self-complementary AAV: CNS: central nervous system; AAV-rh: AAV pseudotype rhesus; ssAAV: single-stranded AAV; BBB: blood–brain barrier; MM: mitochondrial myopathy; PEO: progressive external ophthalmoplegia; KO: knock-out; SDO: sulfur dioxygenase; EMA: ethylmalonic acid; COX: cytochrome c oxidase; MDS: mtDNA depletion syndrome; MNGIE: mitochondrial neurogastrointestinal encephalomyopathy; TP: thymidine phosphorylase; PGK: phosphoglycerate kinase; HLP: hybrid liver-specific promoter; AAT: alpha-1-antitrypsin; dC: deoxycytidine; dT: deoxythymidine; dU: deoxyuridine; KD: ketogenic diet; LS: Leigh syndrome; CMV: cytomegalovirus; IV: intravenous; ICV: intracerebroventricular; FRDA: Friedreich ataxia; LHON: Leber’s hereditary optic neuropathy; MTS: mitochondrial target sequence; MERRF: myoclonic epilepsy with ragged red fibers; KSS: Kearns–Sayre syndrome; ZFNs: zinc-finger nucleases; TALENs: transcription activator-like effector nucleases; DdCBEs: DddA-derived cytosine base editors; TALEDs: TALE-linked deaminases; IUFGT: in utero fetal gene therapy; GGT: germline gene therapy; MMA: methylmalonic acidemia; DSBs: double-strand breaks; MRT: mitochondrial replacement therapy.
